# Assessing Myocardial Strain and Myocardial Work as a Marker for Hypertensive Heart Disease: A Meta-Analysis

**DOI:** 10.31083/j.rcm2408217

**Published:** 2023-07-31

**Authors:** Simon W. Rabkin

**Affiliations:** ^1^Department of Medicine, Division of Cardiology, University of British Columbia, Vancouver, BC V5Z 1M9, Canada

**Keywords:** global left ventricular strain, global longitudinal strain, global circumferential strain, global radial strain, hypertension, left ventricular hypertrophy, global left ventricular work

## Abstract

**Background:**

The main objective of this study was to determine whether myocardial strain and myocardial work are altered in hypertension and whether the strain is independent of hypertension-induced left ventricular hypertrophy.

**Methods:**

Two systematic literature searches were conducted using Medline and EMBASE through to June 30, 2022. In the first, search terms left ventricular strain or speckle tracking AND hypertension and left ventricular hypertrophy were used in conjunction with Boolean operators to identify articles reporting left ventricular strain in patients with hypertension. In the second, the terms Global cardiac or myocardial work AND hypertension were used to identify articles. Publication bias was assessed by examination of funnel plots and calculation of the Failsafe N and Duval and Tweedie’s Trim and fill. The results were presented as Forrest plots.

**Results:**

Global longitudinal strain (GLS) was significantly lower in patients with hypertension compared to those without hypertension with a mean difference of 2.0 ± 0.1 (standard error of mean(SEM)) in the fixed effect model. Global circumferential strain (GCS) was significantly lower in hypertension. The mean difference between the hypertensive and non-hypertensive groups was 1.37 ± 0.17. Global radial strain (GRS) was significantly (*p* < 0.05) greater in hypertension. However, this difference was significant in only 3 and of borderline significance in 3 of 14 studies where GRS was measured. The mean difference between the hypertensive and non-hypertensive groups was 1.5 ± 0.5 using the fixed effects model. There was a significant relationship between GLS and GCS as well as between GCS and GRS but no significant relationship between GLS and GRS. There was no significant difference in left ventricular ejection fraction (LVEF) between the hypertension and no hypertension groups. There was no significant relationship between LVEF and either GLS or GCS but a significant negative correlation was found between LVEF and GRS. GLS was further reduced in persons with hypertension and left ventricular hypertrophy (LVH) compared to hypertension without LVH. In contrast, there were no or minimal differences in GCS and GRS for individuals with hypertension and LVH compared to those without LVH. Global myocardial work index (GWI) and Global constructive work (GCW) were significantly greater in patients with hypertension compared to controls. Global wasted work (GWW) indicated significantly less wasted work in controls compared to hypertension. In contrast, Global work efficiency (GWE) was significantly lower in hypertension compared to the control.

**Conclusions:**

There was a significant reduction in GLS and GCS in hypertension while GRS was increased. The reduction in GLS in hypertension was not dependent on the presence of LVH. GLS was further reduced in persons with hypertension when LVH was present. In contrast, there were no or minimal differences in GCS and GRS for individuals with LVH compared to those without LVH. GLS was independent of left ventricle (LV) ejection fraction. GWI, GCW and GWW were greater in hypertension while GWE was lower in hypertension compared to controls. These data support the contention that GLS and indices of global work are early markers of hypertensive heart disease.

## 1. Introduction

The impact of hypertension on the heart includes thickening of the left ventricular wall that later leads to insufficient myocardial perfusion—myocardial ischemia, and to heart failure with both reductions in systolic and diastolic function [1, 2, 3, 4, 5]. Early recognition of the consequences of hypertension on the heart may be an indication for more vigorous antihypertensive drug treatment to avert or minimize the development of the full blown consequences of hypertension on the heart.

One approach that has attracted recent attention, to identify the early cardiac effects of hypertension is the assessment of myocardial strain, which has proved to be useful in recognizing the early adverse effects of cancer chemotherapeutic agents on the heart. Myocardial strain is a dimensionless index of length change between two given points, which reflects the degree of myocardial deformation [6]. It has been recognized for a long time that the contractile function of the heart is dependent on contraction of myocardial fibers that have different orientations at various levels of the heart [7, 8, 9]. The longitudinal arrangement of fibers on the oblique parts of the heart contrasts with the circumferential arrangement of those on other parts of the heart [8]. Contraction of myocardial fibers that have different orientations produces deformation in different directions so that strain can be assessed in the various directions in which the myocardium deforms. Cardiomyocyte deformation, stretching, shortening and thickening in the different myocardial layers translates into left ventricular stretching, shortening and thickening, that can be measured as percentage longitudinal, circumferential and radial strain [10]. Subendocardial and subepicardial layers are purported to be mainly responsible for longitudinal strain; mid-myocardial layers mainly account for circumferential strain and thickening of all fibers in all three layers is responsible for radial strain [10]. Longitudinal strain evaluates the apex-base deformation, circumferential strain evaluates circumferential deformation while radial strain represents radial thickening of the myocardium [10, 11]. However, the distribution and angulation of myofibers in all layers can contribute to each of these three kinds of strain [10].

Until recently it was not possible to readily assess changes in myocardial contractility in the different orientations in the heart. The introduction of speckle tracking echocardiography permitted a quantitative assessment of myocardial motion in discrete areas of the myocardium that correspond to different layers of the heart [12]. Speckle tracking echocardiography provides accurate and angle-independent measurements of left ventricle (LV) dimensions [13]. There is evidence that assessment of myocardial strain may be superior to the left ventricular ejection fraction as a predictor of major adverse cardiac events such as cardiac death and hospitalization due to heart failure [14, 15].

A relatively new method to evaluate myocardial systolic performance is the concept of assessing myocardial work performed during systole because it takes into account not only left ventricular deformation (strain) but also adjusts for after load which can influence LV strain [16]. The left ventricular pressure-strain relationship can be assessed noninvasively incorporating systemic arterial blood pressure coincident with measurement of left ventricular strain from which several different kinds of myocardial work can be calculated [16, 17]. Global myocardial work indices obtained from LV pressure–strain loop (LV PSL) strongly correlate with invasive measurements [18]. Global myocardial work index (GWI) represents the total work within the area of the LV PSL. Constructive myocardial work (GCW) represents work performed by LV ejection during systole. Global wasted work (GWW) is work performed by the LV that does not contribute to LV ejection. Global work efficiency (GWE) is the ratio of global constructive myocardial work (GCW) to global wasted work (GWW) and represents the efficiency of LV mechanical energy that is expended in systole.

Whether myocardial strain is altered in hypertension and whether it is independent of hypertension-induced left ventricular hypertrophy is an ongoing question. Some investigators concluded that there were no differences in some elements of left ventricular strain in hypertension while other investigators concluded the reverse [19, 20, 21, 22]. The assessment of left ventricular strain in different directions may compound the variability of the results. Whether myocardial work is altered in hypertension is also unresolved. The objectives of this review were several folds to focus on hypertension and determine (i) which type of myocardial strain, longitudinal, circumferential or radial, if any were abnormal in hypertension (ii) whether any abnormality in strain was related to or independent of left ventricular ejection fraction or left ventricular hypertrophy and (iii) whether hypertension alters myocardial work indices.

## 2. Methods

### 2.1 Literature Search

A systematic search was conducted of Medline and EMBASE. The search was conducted from the inception of each database through to June 30, 2022. Search terms left ventricular strain or speckle tracking AND hypertension and left ventricular hypertrophy were used in conjunction with Boolean operators to identify articles reporting left ventricular strain in patients with hypertension. A second search was conducted with the terms myocardial OR cardiac work AND hypertension. Because there was no primary patient or animal contact, there was no requirement for approval from our research ethics committee. The meta-analysis was not registered. The search was conducted according to the Preferred Reporting Items for Systematic Reviews and Meta-Analyses (PRISMA) [23] (**Supplementary Fig. 1**).

Titles and abstracts were screened to identify articles for full-text review. The inclusion criteria included echocardiographic measurement of left ventricular strain or myocardial work. The exclusion criteria were articles: (i) not published in English; (ii) involved non-human subjects; (iii) non-primary research articles (reviews, editorials or letters commenting on an article); (iv) pediatric age population (v) secondary hypertension (vi) unrelated to the investigated topic, e.g., only focused on ECG and ECG pattern of left ventricular hypertrophy (LVH) and strain; and (vii) did not provide a direct comparison of control and individuals with hypertension, i.e., focused only on an aspect of LV strain or work or (viii) relevant data could not be extracted from the paper.

The following items were extracted from each paper, authors, year of publication, age, sex, left ventricular mass, left ventricular ejection fraction, ventricular longitudinal strain, circumferential strain and radial strain as well as indices of myocardial work.

### 2.2 Statistical Analysis

Results were quantified using forest plots depicting the standard difference of means, 95% confidence interval, and *p*-value. The meta-analysis was performed using Comprehensive Meta-Analysis (Biostat Inc., NJ, USA). Study heterogeneity in the meta-analysis was tested using Cochran’s Q, $I^{2}$ statistic and ${\mathrm{Tau}}^{2}$ where variance is described by SEM. Otherwise the data is presented as the mean ± SD. Publication bias was assessed by examination of funnel plots and calculation of the failsafe N and Duval and Tweedie’s Trim and fill.

## 3. Results

The initial search for left ventricular strain produced 56 references after the elimination of duplicates. After filtering the titles and abstracts, 9 were eliminated because they were review articles, editorials or letters. The full text review eliminated 32 reports, and 4 articles were added by ‘hand searching’ and examination of bibliographies of existing papers, eventually, 19 articles could be included in the systematic review. 18 studies had a control group and one study compared patients with hypertension with and without left ventricular hypertrophy [19, 20, 21, 22, 24, 25, 26, 27, 28, 29, 30, 31, 32, 33, 34, 35, 36, 37, 38] (**Supplementary Fig. 1**). The initial search for myocardial work and hypertension produced 107 references after the elimination of duplicates. After filtering the titles and abstracts, 19 were eliminated because they were review articles, editorials or letters and 6 were eliminated because they were animal studies. Seventy-three were eliminated because they were not relevant, most of them because they were published before the current form of assessment and classification of non-invasive assessment of myocardial work. Nine studies were subjected to meta-analysis [39, 40, 41, 42, 43, 44, 45, 46, 47]. A similar summary for data evaluation considering global work indices was conducted (**Supplementary Fig. 2**).

For studies of left ventricular strain, the patient characteristics of the studies demonstrate a range of mean ages, from 29 to 70 years (Table 1, Ref. [19, 20, 21, 22, 24, 25, 26, 27, 28, 29, 30, 31, 32, 33, 34, 35, 36, 37, 38]). The majority of studies had a mean age in the 50 years age group. The sex distribution also varied between studies from 15% to 100% with most studies having a majority of men. Studies were separated into those that had a control group and those that compared patients who did or did not have left ventricular hypertrophy. The degree of left ventricular hypertrophy was included. For studies of myocardial work, the patient characteristics of the studies demonstrate a range of mean ages, from 38 to 72 years (Table 2, Ref. [39, 40, 41, 42, 43, 44, 45, 46, 47]). Assessment of the quality of studies is challenging for non-randomized case control studies especially the type of studies that comprise the data base for this meta-analysis [48]. The most frequently used assessment methodology—the Newcastle-Ottawa scale was applied and ranked all of the studies low. That scale gives a lower rank to studies with (i) hospital based groups compared to population-based studies; (ii) no intervention in the case groups that could be graded and (iii) lack of details to evaluate accurate matching procedures for all variables in the controls [48]. While the grading system ranked the studies low, it is a characteristic of the nature of all of the ranking systems but importantly the ratings were consistent between studies which justifies the inclusion of all studies in this analysis. Other assessment methods such as QUADAS rely on a grading of the reference standard and disease progression bias which are not relevant for the kinds of studies in this review [49].

**Table 1.** **The patient characteristics for the studies of left ventricular strain**.

Author	Assessment	Control					HTN				
		N	Age (yrs)	Sex (%M)	LV mass (g/$m^{2}$)	LVEF	N	Age (Yrs)	Sex (%M)	LV mass (g/$m^{2}$)	LVEF
Lembo *et al*. 2020 [22]	echocardiogram +	115	42	60	31	62.6	180	44	63	33	62.5
Esposito *et al*. 2019 [20]	echocardiogram	82	54	59	88	60.4	18	56	50	101	58.7
Xu *et al*. 2019 [36]	echocardiogram	50	53	24	84	64.4	80	54	50	131	65.1
Sun *et al*. 2019 [38]	echocardiogram	80	51	79	92	65	80	51	79	116	64.3
Mordi *et al*. 2018 [28]	echocardiogram	28	68	50	NA	64.3	22	67	77	NA	65.6
Minatoguchi *et al*. 2017 [29]	echocardiogram	54	69	61	87	67	50	70	60	95	67.9
Huang *et al*. 2016 [37]	echocardiogram	42	50	NA	NA	65	63	55	NA	NA	64.6
Szelenyi *et al*. 2015 [30]	ecocardiogram	18	66	33	90	67.6	94	69	34	109	65.3
Santoro *et al*. 2014 [19]	echocardiogram	17	40	100	89	58.7	22	48	100	126	58.5
Shin *et al*. 2014 [35]	echocardiogram	40	29	78	79	62.1	40	30	84	84	62
Tadic *et al*. 2014 [34]	echocardiogram	49	49	78	39	60	50	48	80%	48	56.6
Celic *et al*. 2014 [27]	echocardiogram +	50	46	76	39	65	152	48	77	45	63.3
Ozkan *et al*. 2014 [26]	echocardiogram	40	52	70	66	66.2	78	51	65	102	65.1
Kouzu *et al*. 2011 [31]	echocardiogram	55	59	15	83	66	74	62	23	114	66.4
Imbalzano *et al*. 2011 [33]	echocardiogram	51	52	65	64	63	51	51	65	103	59.5
Galderisi *et al* 2010 [32]	echocardiogram +	19	29	100	31	61.7	18	33	100	37	61.6
Cappelli *et al*. 2009 [24]	echocardiogram	24	45	54	93	62.7	22	52	77	140	64.1
Narayanan *et al*. 2009 [21]	echocardiogram	52	49	27	66	66	52	53	46	89	67.9
		HTN no LVH					HTN with LVH				
		N	Age	Sex m%	LV mass (g/$m^{2}$)		N	Age	sex m%	LV mass (g/$m^{2}$)	
Esposito *et al*. 2019 [20]	echocardiogram	18	56	56	81		10	58	0.9	137	
Xu *et al*. 2019 [36]	echocardiogram	40	53	50	86		40	55	50	126	
Minatoguchi *et al*. 2017 [29]	echocardiogram	50	70	70	60		40	69	58	132	
Huang *et al*. 2016 [37]	echocardiogram	35	55	NA	NA		28	54	NA	NA	
Szelenyi *et al*. 2015 [30]	echocardiogram	38	66	24	105		56	72	41	125	
Ozkan *et al*. 2014 [26]	echocardiogram	38	49	63	81		40	53	68	123	
Goebel al 2011 [25]	echocardiogram +	36	65	44	42		44	63	38	66	
Imbalzano *et al*. 2011 [33]	echocardiogram	24	53	58	81		27	60	70	122	
	Echocardiogram+ is g/$m^{2.7}$										

HTN, hypertension; LV, left ventricle; LVEF, left ventricular ejection fraction; LVH, left ventricular hypertrophy; NA, not available. + is the symbol that refers to different units of LVH.

**Table 2.** **The patient characteristics for the study of left ventricular work**.

Author	Control					HTN				
	N	Age (yrs)	Sex (%M)	LV mass (g/$m^{2}$)	LVEF (%)	N	Age	Sex (%M)	LV mass (g/$m^{2}$)	LVEF (%)
Tsai *et al*. 2022 [39]	32	53	37.5	74	70	43	51	56	74	67
de Andrade Hygidio *et al*. 2022 [40]	16	61	35	83	66	55	61	25	91	66
Ding *et al*. 2022 [41]	40	49	NA	91	67	60	58	NA	93	66
Huang *et al*. 2021 [42]	53	47	53	94	65	95	49	62	107	64
Tadic *et al*. 2021 [44]	45	54	53	67	61	159	56	56	83	60
Jaglan *et al*. 2021 [45]	15	38	47	77	60	65	65	46	97	61
Loncaric *et al*. 2020 [46]	30	54	44	68	58	139	57	52	76	56
Tadic *et al*. 2020 [43]	55	51	52	70	63	110	55	52	87	62
Chan *et al*. 2019 [47]	8	54	38	77	61	37	72	65	186	62

HTN, hypertension; LV, left ventricle; LVEF, left ventricular ejection fraction.

**Fig. 1.**
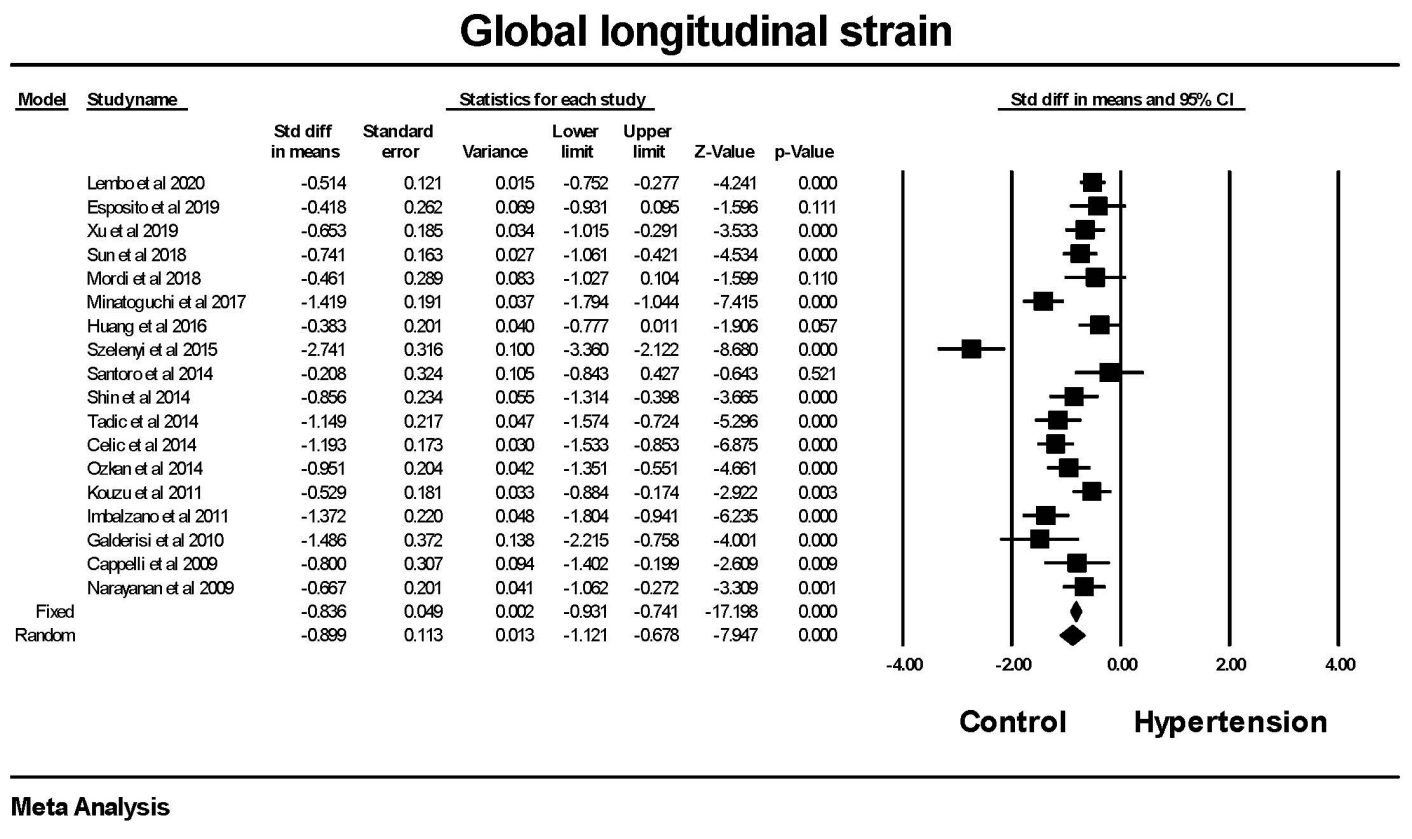
**The forest plot for Global longitudinal strain (GLS) in patients with hypertension compared to control groups without hypertension**. The overall standard difference of the means was significant for both the fixed and random effects models. There was significant heterogeneity with Q = 99.8, *p* < 0.001, $I^{2}$ = 83.0 and ${\mathrm{Tau}}^{2}$ = 0.689 ± 0.313 (SEM). The failsafe N was 1201 and Duval and Tweedie’s Trim and fill was –0.772 (–0.867, –0.677) for the fixed effect model. SEM, standard error of mean; CI, confidence interval.

Global longitudinal strain was significantly lower in patients with hypertension compared to those without hypertension (Fig. 1). The majority, 13 of the 18 studies, showed a significant difference between hypertension and control group. The mean difference between the hypertensive and non-hypertensive groups was 2.0 ± 0.1 (SEM) in the fixed model and 2.1 ± 0.3 in the random effects model, although there was a significant amount of heterogeneity between studies. There was a low probability for publication bias. The failsafe N was 1201 or one would have to find 1201 null studies for the relationship between hypertension and GLS to be not significant (a 2 tailed *p* > 0.05).

**Fig. 2.**
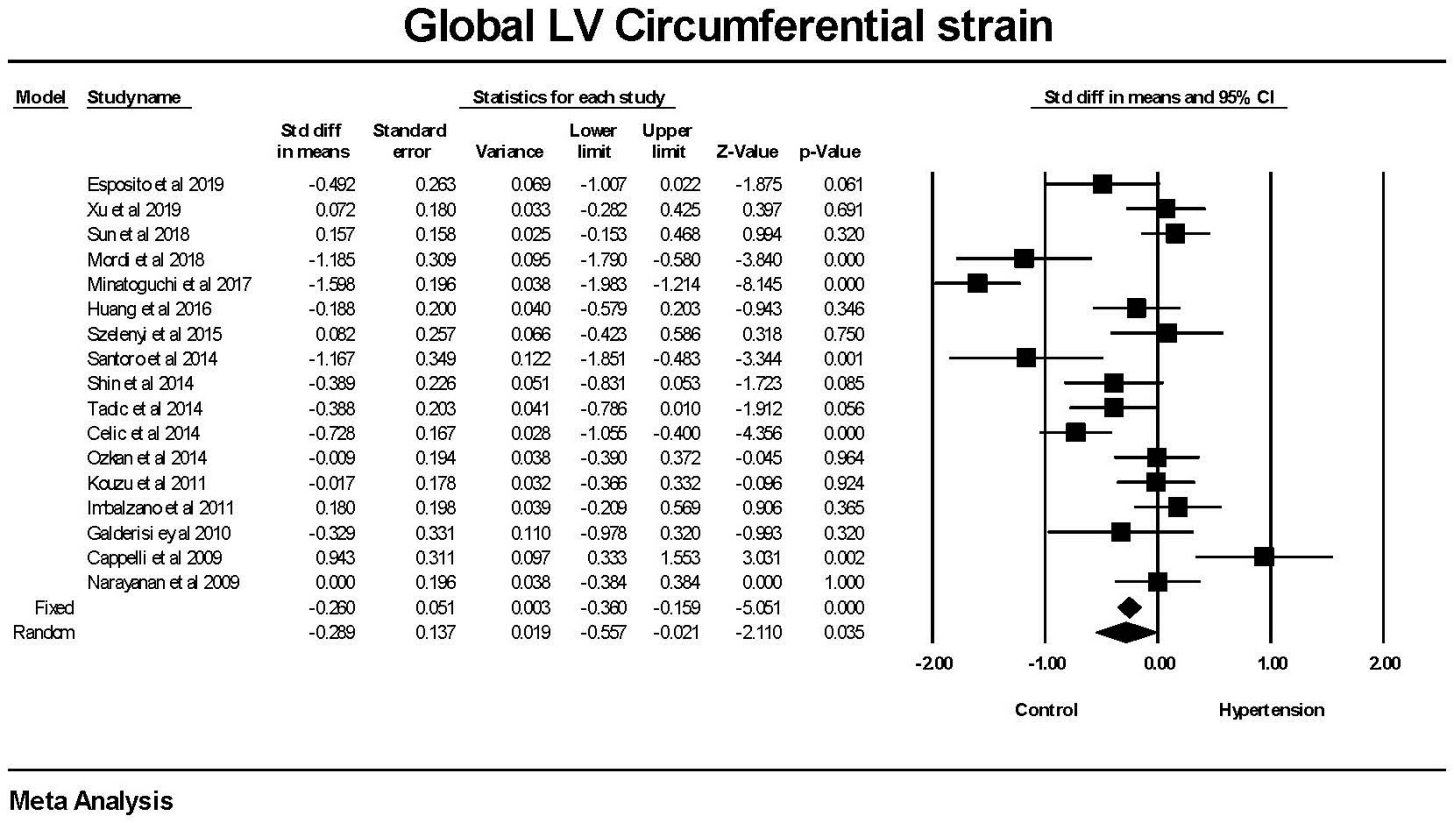
**The forest plot for Global Circumferential strain (GCS) in patients with hypertension compared to control groups without hypertension**. The overall standard difference of the means was significant for both the fixed and random effects models. There was significant heterogeneity with Q = 109.015, *p* < 0.001, $I^{2}$ = 85.323 and ${\mathrm{Tau}}^{2}$ = 0.264 ± 0.116 (SEM). The failsafe N was 105 and Durval and Tweedie’s Trim and fill was –0.260 (–0.80360, –0.159) for the fixed effect model. LV, left ventricle; CI, confidence interval.

Global circumferential strain was significantly lower in patients with hypertension compared to those without hypertension (Fig. 2). There was a significant amount of heterogeneity between studies. The mean difference between the hypertensive and non-hypertensive groups was 1.37 ± 0.17 (SEM) using the fixed effects model and 0.87 ± 0.45 in the random effects model. The failsafe N was 105 or one would have to find 105 null studies for the relationship between hypertension and GLS to have a 2 tailed *p* > 0.05.

**Fig. 3.**
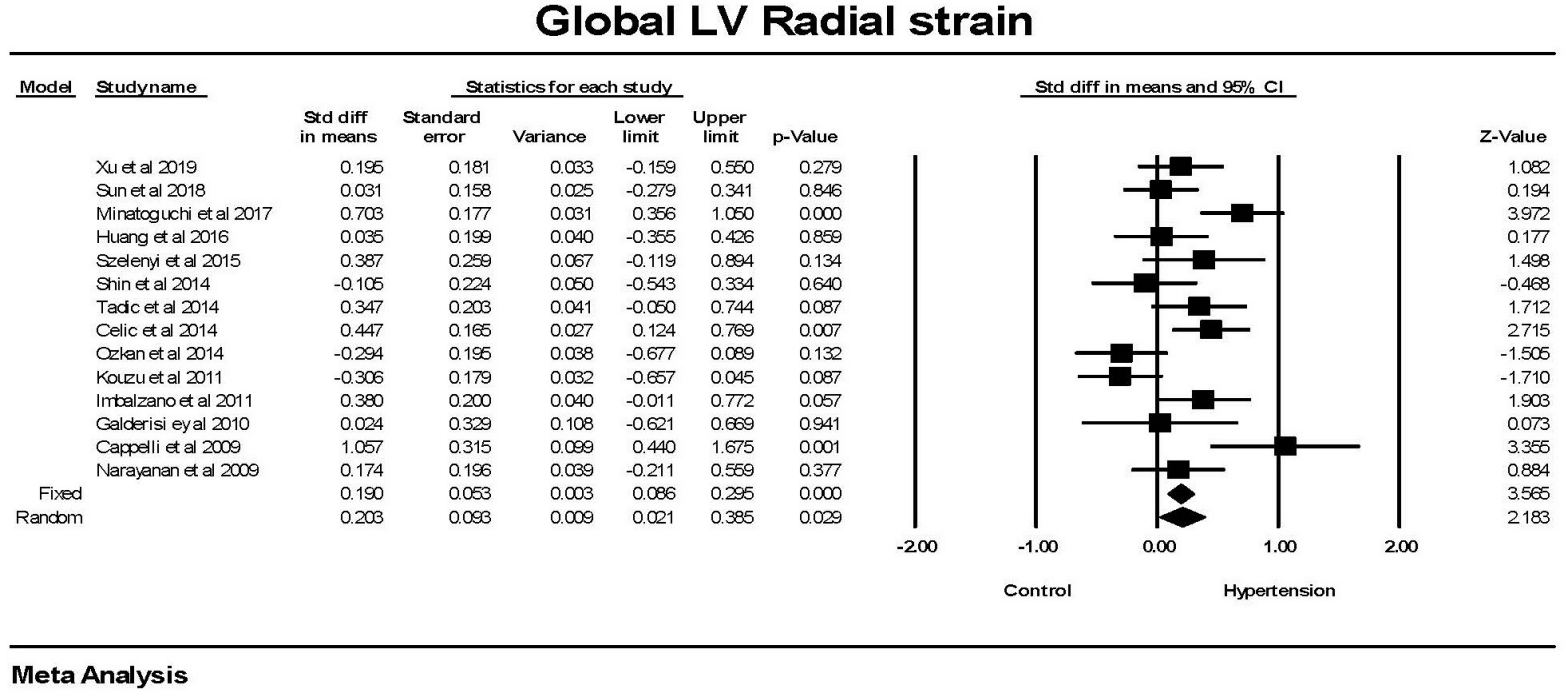
**The forest plot for Global radial strain (GRS) in patients with hypertension compared to control groups without hypertension**. There was significant heterogeneity with Q = 37.9, *p* < 0.001, $I^{2}$ = 65.7 and ${\mathrm{Tau}}^{2}$ = 0.077 ± 0.47 (SEM). The failsafe N was 37 and Duval and Tweedie’s Trim and fill was 0.190 (0.086, 0.295) for the fixed effect model. LV, left ventricle; CI, confidence interval.

Global radial strain was significantly (*p* < 0.05) greater in patients with hypertension compared to those without hypertension (Fig. 3). However, this difference was significant in only 3 studies and was of borderline significance in 3 of 14 studies where GRS was measured. There was a significant amount of heterogeneity between studies. The mean difference between the hypertensive and non-hypertensive groups was 1.5 ± 0.5 using the fixed effects model and 2.3 ± 1.0 (SEM) using the random effects model. The failsafe N was 37 or one would have to find 37 null studies for the relationship between hypertension and GRS to have a 2 tailed *p* > 0.05.

**Fig. 4.**
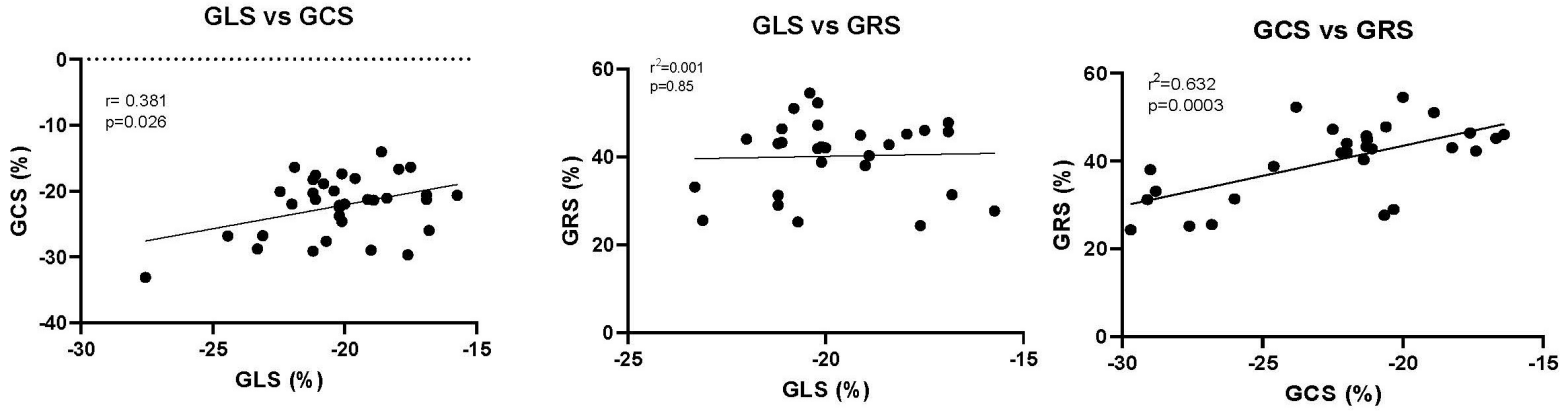
**The relationship between GLS, GCS and GRS**. Each point represents the mean value for both the control and the individuals with hypertension from each study. The Pearson’s r correlation and the *p* value are shown. GLS, Global longitudinal strain; GCS, Global circumferential strain; GRS, Global radial strain.

In the entire population, control and hypertensive group, there was a significant relationship between GLS and GCS as well as between GCS and GRS (Fig. 4). There was no significant relationship between GLS and GRS.

**Fig. 5.**
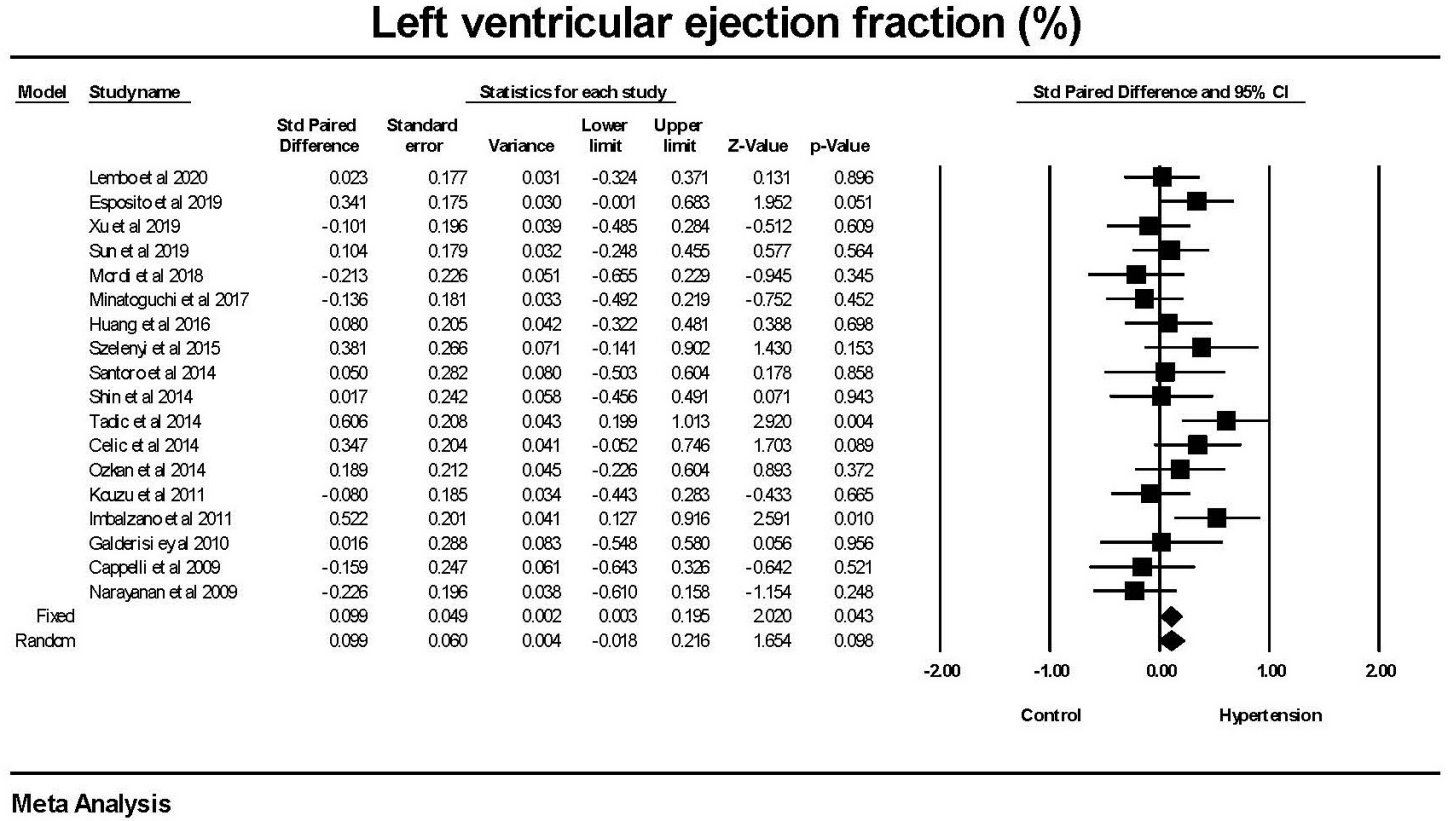
**The forest plot for left ventricular ejection fraction (LVEF) in patients with hypertension compared to control groups without hypertension**. There was a weak difference in LVEF in the fixed effect model (*p* = 0.043) but none in the random effects model (*p* = 0.098). There was no significant heterogeneity with Q = 24.9, *p* = 0.097, $I^{2}$ = 31.7 and ${\mathrm{Tau}}^{2}$ = 0.02 ± 0.022 (SEM). CI, confidence interval; SEM, standard error of mean.

There was no significant difference in the left ventricular ejection fraction between the hypertension and non-hypertension groups (Fig. 5).

**Fig. 6.**
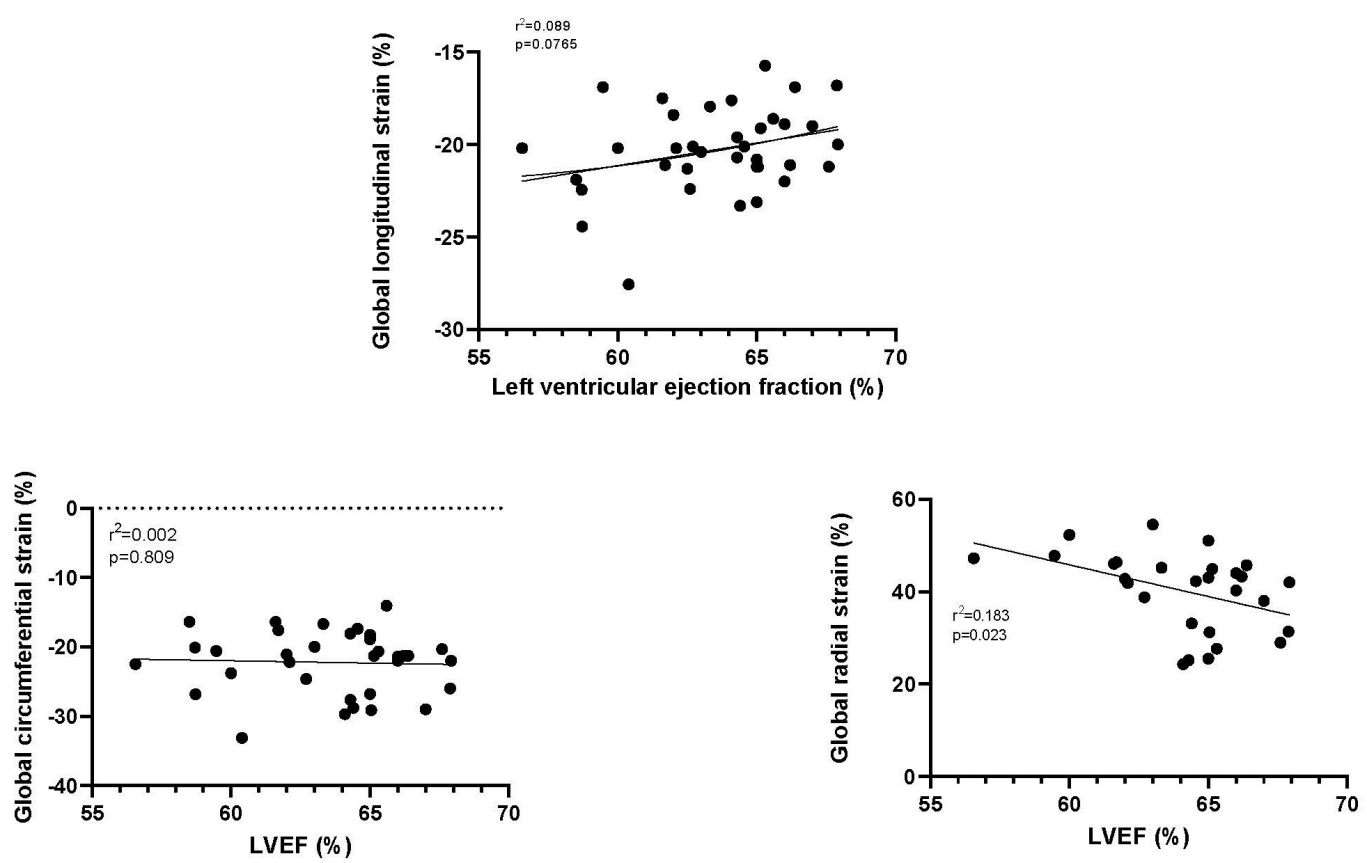
**The relationship between left ventricular ejection fraction (LVEF) and GLS, GCS and GRS**. GLS, Global longitudinal strain; GCS, Global circumferential strain; GRS, Global radial strain.

Examining the relationship between LVEF and global strain did not find any significant relationships between LVEF and either GLS or GCS but a significant negative correlation between LVEF and GRS (Fig. 6).

In order to explore whether left ventricular hypertrophy impacted the changes in left ventricular strain, an analysis was conducted on the subset of studies that evaluated LV strain in patients with hypertension with and without LVH (Table 3, Ref. [20, 25, 26, 29, 30, 33, 36, 37]). Most, but not all, studies, used the same definition of LVH.

**Table 3.** **The formula used to calculate left ventricular mass and the criteria for definition of left ventricular hypertrophy**.

Author	Criterion	Definition left ventricular hypertrophy
Esposito *et al*. 2019 [20]	European Society of Cardiology guidelines.	Not defined
Xu *et al*. 2019 [36]	Devereux formula	≥115 g/$m^{2}$ in men and ≥95 g/$m^{2}$ in women
Minatoguchi *et al*. 2017 [29]	American Society of Echocardiography recommendations	>115 g/$m^{2}$ in men and >95 g/$m^{2}$ in women
Huang *et al*. 2016 [37]	Not defined	>125 g/$m^{2}$ in men and >110 g/$m^{2}$ in women
Szelenyi *et al*. 2015 [30]	Devereux formula	≥115 g/$m^{2}$ in men and ≥95 g/$m^{2}$ in women
Ozkan *et al*. 2014 [26]	Devereux formula	≥115 g/$m^{2}$ in men and ≥95 g/$m^{2}$ in women
Goebel al 2011 [25]	Devereux formula	Not defined
Imbalzano *et al*. 2011 [33]	American Society of Echocardiography recommendations	>102 g/$m^{2}$ in men and >81 g/$m^{2}$ in women

**Fig. 7.**
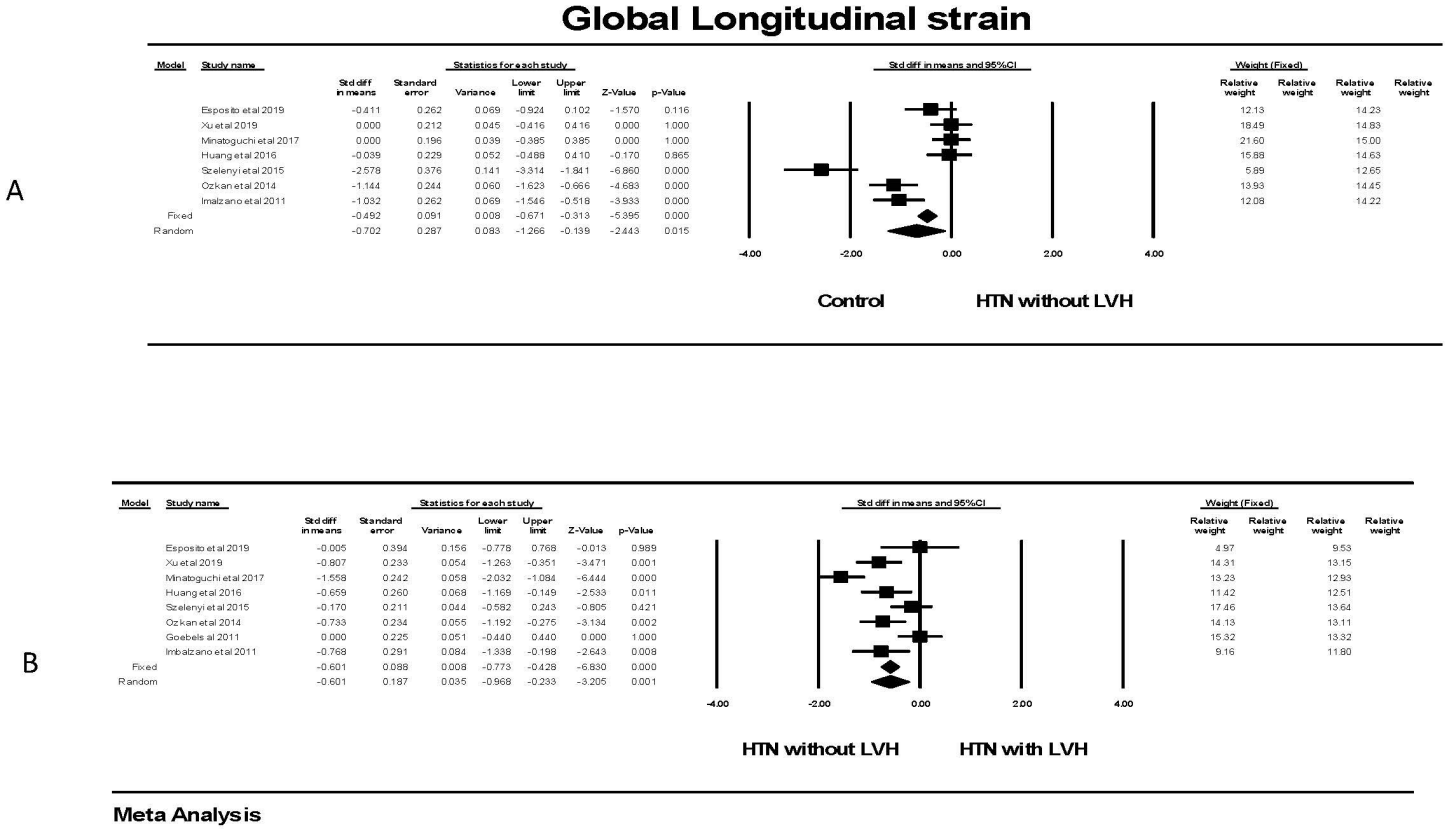
**Global longitudinal strain in patients with hypertension**. (A) shows the forest plot for Global longitudinal strain (GLS) in patients with hypertension but no left ventricular hypertrophy compared to control groups without hypertension. There was significant heterogeneity with Q = 59.5, *p* < 0.001 $I^{2}$ = 89.6 and ${\mathrm{Tau}}^{2}$ = 0.512 ± 0.343 (SEM). The weighting of studies in each of the two models is shown on the right side of the figure. The failsafe N was 76 and Duval and Tweedie’s Trim and fill was –0.513 (–0.8689, –0.338) for the fixed effect model. (B) shows the forest plot for Global longitudinal strain (GLS) in patients with hypertension but no left ventricular hypertrophy compared to individuals with hypertension and LVH. There was significant heterogeneity with Q = 30.8, *p* < 0.001 $I^{2}$ =77.2 and ${\mathrm{Tau}}^{2}$ = 0.213 ±0.151 (SEM). The failsafe N was 313 and Derval and Tweedie’s Trim and fill was –1.096 (–1.28, –0.915) for the fixed effect model. CI, confidence interval; HTN, hypertension; LVH, left ventricular hypertrophy; SEM, standard error of mean.

There was a significant (*p* < 0.05) difference in GLS in patients with hypertension and no left ventricular hypertrophy compared to the control groups without hypertension (Fig. 7). This was mainly evident in three of the seven studies. There was significant heterogeneity between those studies. The failsafe N was 76 or one would have to find 76 null studies for the relationship between hypertension and GLS to have a 2 tailed *p* > 0.05. There was also a significant (*p* < 0.05) difference in GLS in patients with hypertension and LVH compared to individuals with hypertension without LVH; a finding that was evident in five of eight studies. There was still heterogeneity between studies but less than comparing the control to hypertension without LVH groups. The failsafe N was 313 or one would have to find 313 null studies for the relationship between hypertension and GLS to have a 2 tailed *p* > 0.05.

Considering both groups, i.e., those with and without hypertension together, there was no correlation between LV mass and GLS (r = 0.117, *p* = 0.522).

**Fig. 8.**
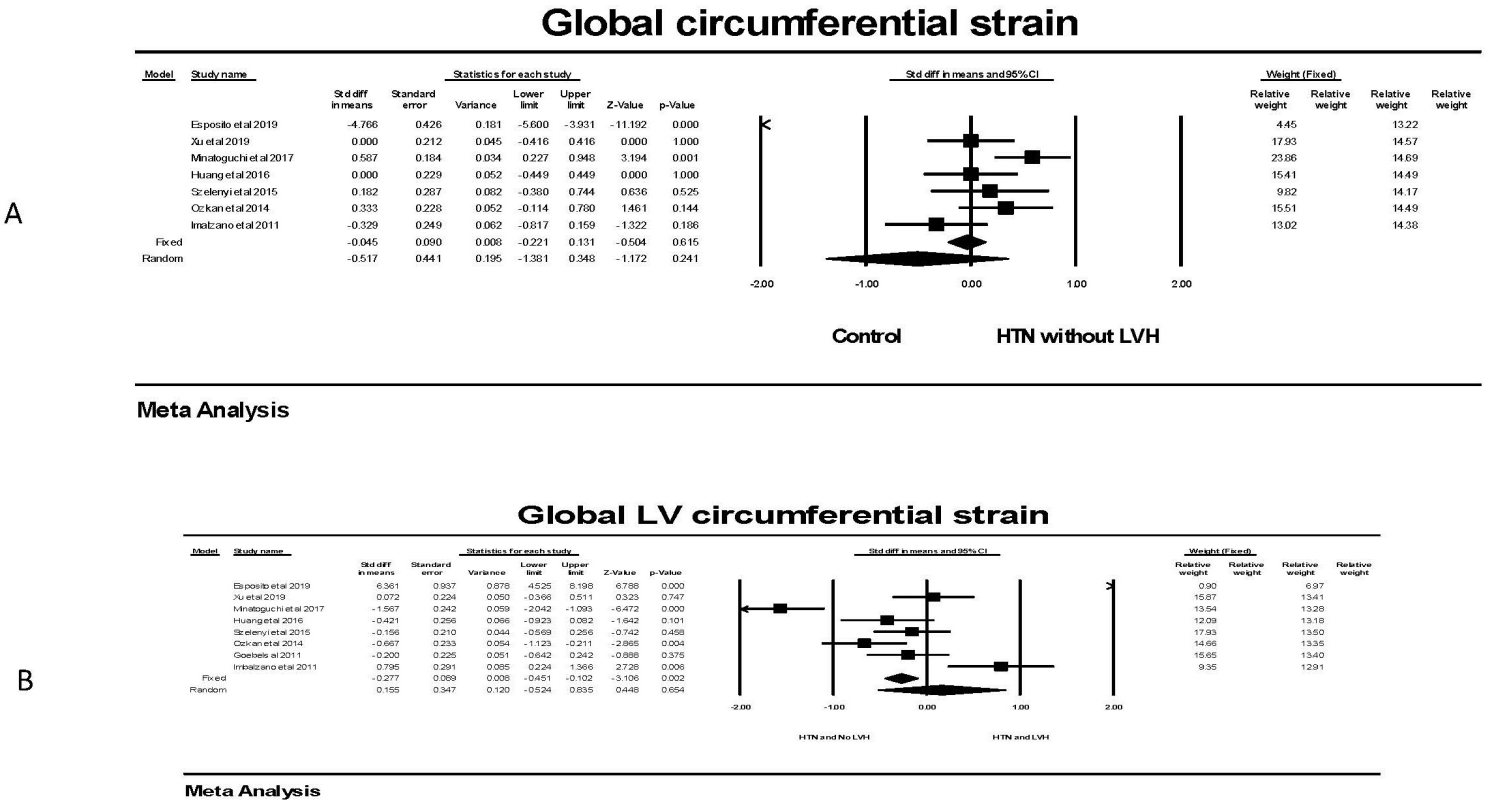
**The forest plots for Global LV circumferential strain and LVH**. (A) shows the forest plot for global circumferential strain (GCS) in patients with no hypertension (HTN) compared to the group with hypertension without left ventricular hypertrophy (LVH). There was significant heterogeneity with Q = 139.5, *p* < 0.001, $I^{2}$ = 95.7 and ${\mathrm{Tau}}^{2}$ = 1.29 ± 0.83 (SEM). The relative weights are shown on the extreme right with the first column for the fixed model and the second column for the random effects model. (B) shows the forest plot for global circumferential strain (GCS) in patients with hypertension but no left ventricular hypertrophy (LVH) compared to individuals with hypertension and LVH. There was significant heterogeneity with Q = 98.1, *p* < 0.001 $I^{2}$ = 92.9 and ${\mathrm{Tau}}^{2}$ = 0.846 ± 0.522 (SEM). CI, confidence interval; HTN, hypertension; LVH, left ventricular hypertrophy; SEM, standard error of mean.

Evaluating global circumferential strain, there was no significant difference in GCS for individuals with hypertension and no LVH compared to individuals without hypertension (Fig. 8). This was found in all studies. There was also no significant difference in the random effects model in persons with hypertension and LVH compared to those with hypertension and no LVH. There was significant heterogeneity in these studies with three showing a significantly lower and one with a significantly higher GCS.

**Fig. 9.**
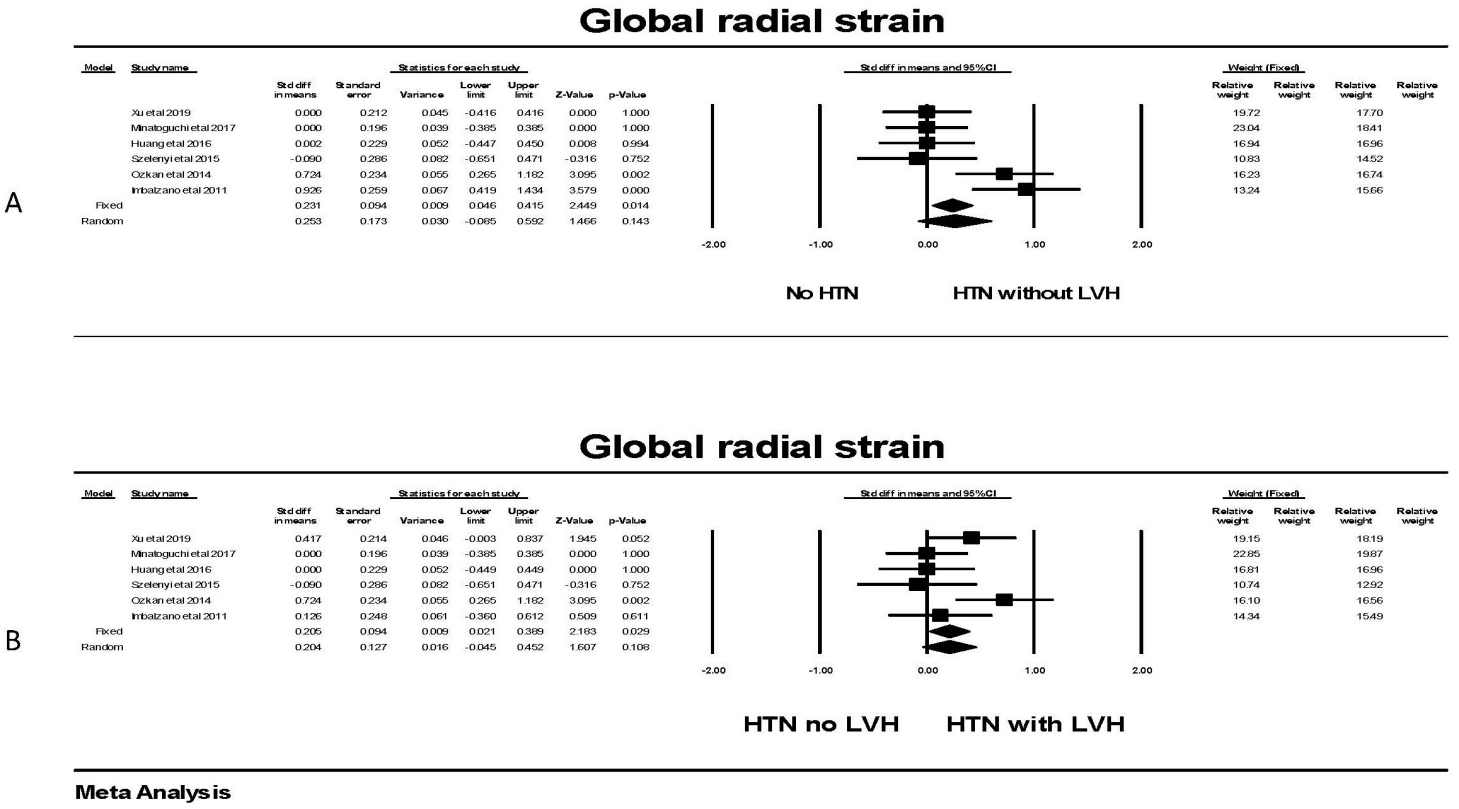
**The forest plot for global radial strain (GRS) in patients with hypertension**. (A) shows the forest plot for global radial strain (GRS) in patients with no hypertension (HTN) compared to the group with hypertension without left ventricular hypertrophy (LVH). There was significant heterogeneity with Q = 16.5, *p* = 0.005, $I^{2}$ = 69.6 and ${\mathrm{Tau}}^{2}$ = 0.125 ± 0.114 (SEM). The relative weights are shown on the extreme right with the first column for the fixed model and the second column for the random effects model. (B) shows the forest plot for global radial strain (GRS) in patients with hypertension (HTN) and no left ventricular hypertrophy (LVH) compared to the group with hypertension with LVH. There was no significant heterogeneity with Q = 8.9, *p* = 0.111, $I^{2}$ = 44.2 and ${\mathrm{Tau}}^{2}$ = 0.042 ± 0.06 (SEM). The relative weights are shown on the extreme right with the first column for the fixed model and the second column for the random effects model. CI, confidence interval; HTN, hypertension; LVH, left ventricular hypertrophy; SEM, standard error of mean.

For global radial strain, there was no significant difference in GRS between individuals without LVH compared to those with LVH in the random effects model (Fig. 9), although a significant difference was found in the fixed effect model; attributable to two studies. Comparing individuals with hypertension, the presence of LVH was not associated with a significant difference in the random effects model although a significant one was observed with the fixed effect model attributable to only one study.

**Fig. 10.**
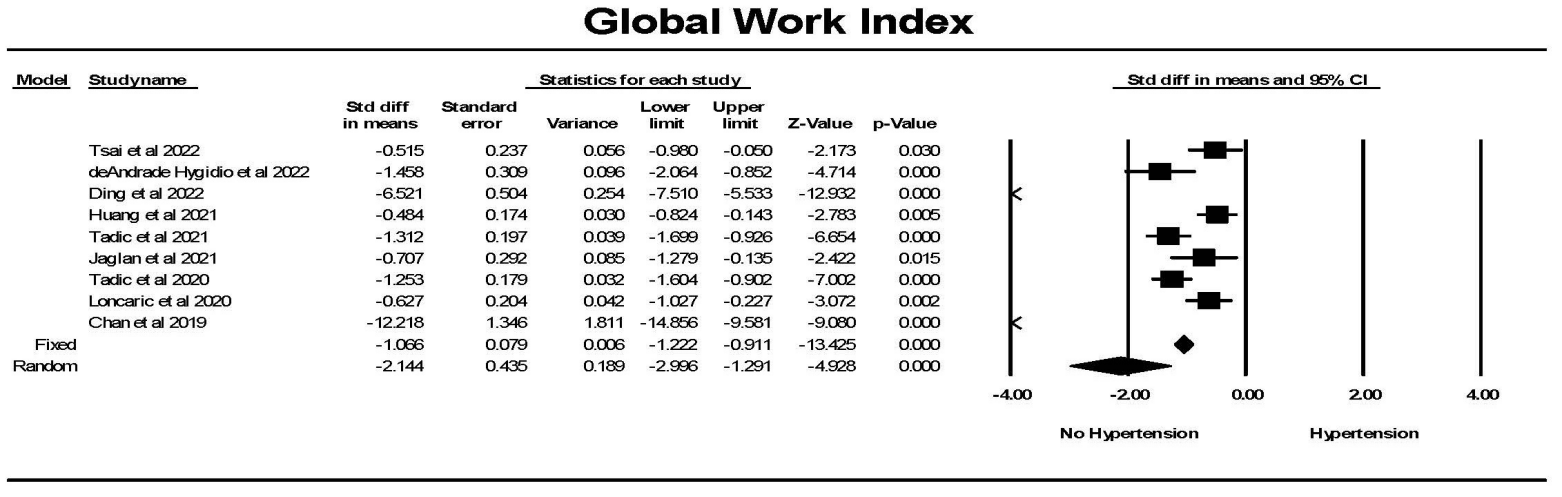
**The forest plot for global myocardial work index (mmHg%) (GWI) in patients with no hypertension (Control) compared to the group with hypertension**. There was significant heterogeneity with Q = 212.7, *p* < 0.0001, $I^{2}$ = 96.3 and ${\mathrm{Tau}}^{2}$ = 0.152 ± 0.91 (SEM). CI, confidence interval; SEM, standard error of mean.

The Global myocardial work index was significantly lower in individuals with hypertension compared to controls (Fig. 10). While there was significant heterogeneity between studies, each study found a significant difference between the groups. Hedges’ g is a measure of effect size which indicates how much the groups differ from one another. Comparing studies that had a concomitant measurement of GLS, Hedges’ g was larger for GWI comparing hypertension to control than for GLS comparing hypertension to control (1.060 ± 0.079 [SEM] vs 0.692 ± 0.056, fixed effects model).

**Fig. 11.**
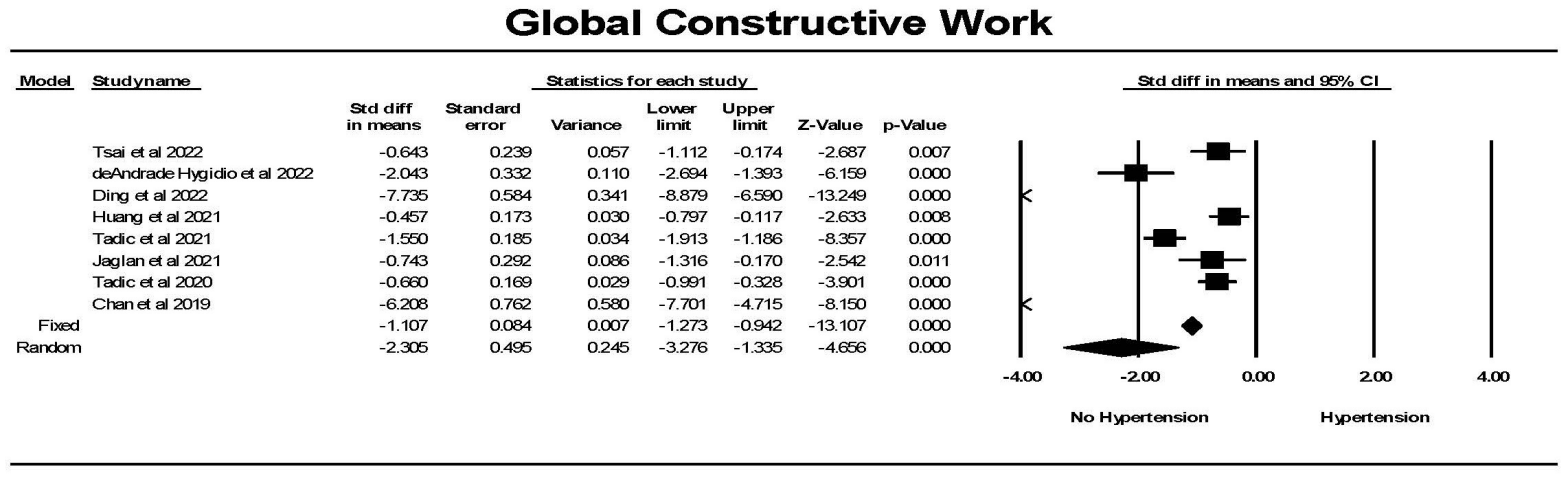
**The forest plot for global constructive work (mmHg%) (GCW) in patients with no hypertension (Control) compared to the group with hypertension**. The arrow represents confidence interval extending beyond the scale. There was significant heterogeneity with Q = 213.8, *p* ≤ 0.0001, $I^{2}$ = 96.7 and ${\mathrm{Tau}}^{2}$ = 1.82 ± 1.23 (SEM). The failsafe N was 584 and Duval and Tweedie’s Trim and fill was –1.107 (–1.272, –0.942) for the fixed effect model. CI, confidence interval; SEM, standard error of mean.

Global constructive work (GCW) was significantly lower in controls than in individuals with hypertension (Fig. 11). While there was significant heterogeneity between studies, each study found a significant difference between the groups. Hedges’ g was larger for GWI comparing hypertension to control than for GLS comparing hypertension to control (1.101 ± 0.084 vs 0.692 ± 0.056, fixed effects model).

**Fig. 12.**
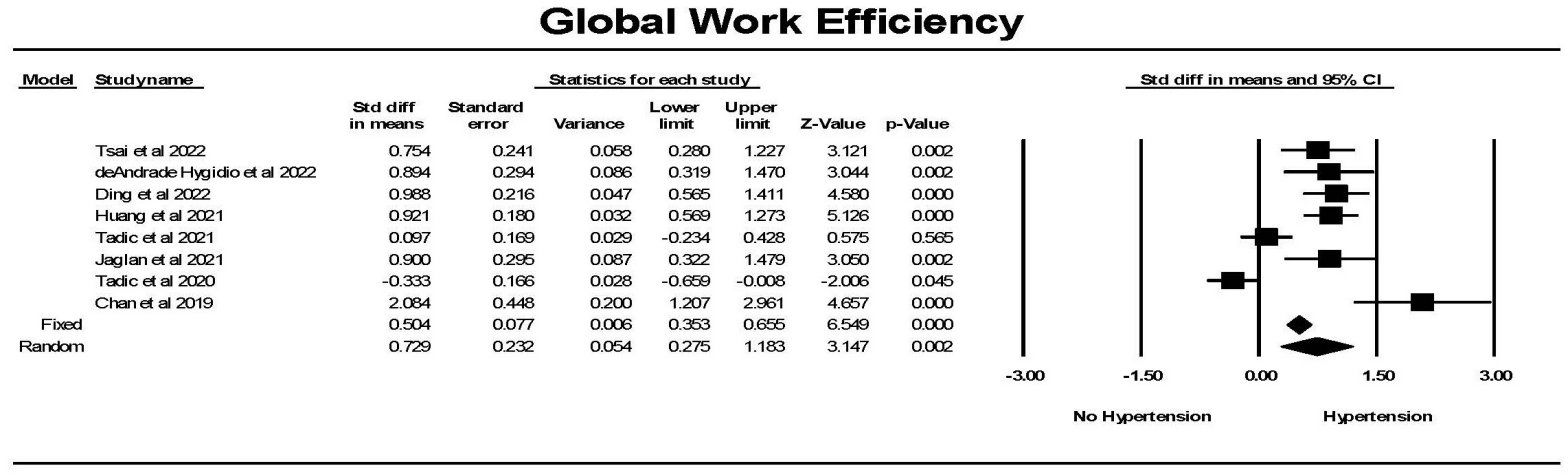
**The forest plot for global work efficiency (GWE) (%) in patients with no hypertension (Control) compared to the group with hypertension**. There was significant heterogeneity with Q = 58.7, *p* < 0.0001, $I^{2}$ = 80.1 and ${\mathrm{Tau}}^{2}$ = 0.364 ± 0.242 (SEM). The failsafe N was 120 and Duval and Tweedie’s Trim and fill was 0.504 (0.350, 0.655) for the fixed effect model. CI, confidence interval; SEM, standard error of mean.

Global work efficiency (GWE) was significantly different in hypertension compared to the control group (Fig. 12). There was one study in which this was not the case and one study where the reverse was found, resulting in considerable heterogeneity between studies. Overall a significant difference between the groups was found. Hedges’ g was smaller for GWI comparing hypertension to control than for GLS comparing hypertension to control (0.502 ± 0.076 vs 0.692 ± 0.056, fixed effects model).

**Fig. 13.**
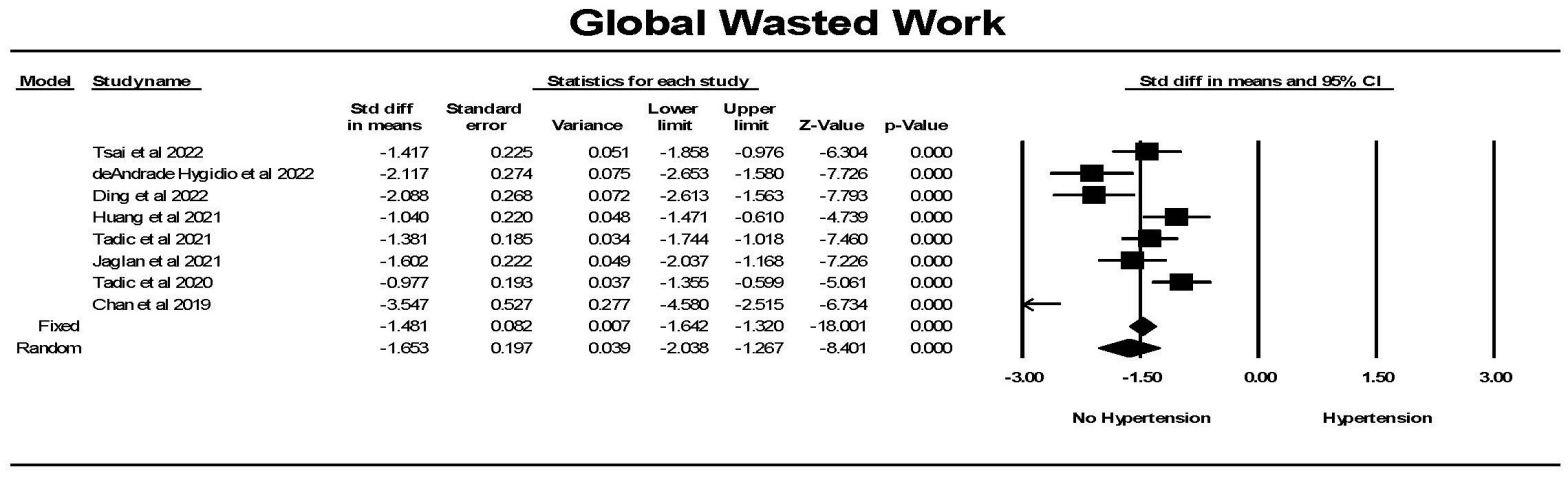
**The forest plot for global wasted work index (mmHg%) (GWW) in patients with no hypertension (Control) compared to the group with hypertension**. The arrow represents confidence interval extending beyond the scale. There was significant heterogeneity with Q = 37.4, *p* < 0.001, $I^{2}$ = 81.3 and ${\mathrm{Tau}}^{2}$ = 0.241 ± 0.167 (SEM). The failsafe N was 725 and Duval and Tweedie’s Trim and fill was –1.481 (–1.642,–1.320) for the fixed effect model. CI, confidence interval; SEM, standard error of mean.

For global wasted work (GWW), there was significantly less wasted work in controls compared to hypertension (Fig. 13). Although there was significant heterogeneity between studies but each study found a significant difference between the groups. Hedges’ g was larger for GWW comparing hypertension to control than for GLS comparing hypertension to control (1.472 ± 0.082 vs 0.692 ± 0.056, fixed effects).

## 4. Discussion

This review concluded that there was a significant reduction in GLS and GCS in hypertension while GRS was increased in hypertension. GLS and GCS were significantly related, with GCS and GRS being strongly associated while GLS and GRS were not significantly related. There was a minimal reduction in LVEF in hypertension. There was no correlation between LVEF and GLS or GCS but a significant negative relationship was noted between LVEF and GRS. The reduction in GLS in hypertension was not dependent on the presence of LVH as GLS was significantly reduced in individuals with hypertension but without LVH compared to individuals without hypertension. However, GLS was further reduced in persons with hypertension and LVH compared to those with hypertension without LVH. In contrast, there were no or minimal differences in GCS and GRS for individuals with LVH compared to those without LVH. The various aspects of myocardial work showed significant differences between persons with hypertension and individuals without hypertension.

In the hypertensive and the control populations, GLS and GCS as well as GCS and GRS were significantly related. In contrast, GLS and GRS were not significantly related. These data suggest that there is some commonality of myocardial fibers and their location within the myocardium while there are distinct differences in the nature of the strain that is being measured when one assessing GLS, GCS or GRS. Subendocardial and subepicardial layers are purported to be mainly responsible for longitudinal strain; mid-myocardial layers mainly account for circumferential strain and thickening of all fibers in all three layers is mainly responsible for radial strain [10]. The distribution and angulation of myofibers in all layers, however, contributes to each of these three kinds of strain [10].

Hypertension was associated with a reduction in GLS, i.e., the deformation from the apex to base was smaller in hypertension. The reduction in GLS is a reflection of a reduction in cardiac contractility. This finding was highly significant in the studies by Lembo *et al*. [22], Xu *et al*. [36], Sun *et al*. [38], Minatoguchi *et al*. [29], Szelenyi *et al*. [30], Shin *et al*. [35], Tadic *et al*. [34], Celic *et al*. [27], Ozkan *et al*. [26], Kouzu *et al*. [31], Imbalzano *et al*. [33], Galderisi *et al*. [32], Cappelli *et al*. [24] and Narayanan *et al*. [21]. There was a minority of studies that did not show a reduction in GLS in hypertension.

Hypertension was associated with a reduction in GCS. Because GCS evaluates the change in circumferential deformation, a smaller GCS is an indicator of a reduction in cardiac contractility. This finding was highly significant in the studies by Mordi *et al*. [28], Minatoguchi *et al*. [29], Santoro *et al*. [19], Celic *et al*. [27], and Cappelli *et al*. [24]. In contrast to GLS, the significant finding with GCS was present in a minority of the 17 studies.

Hypertension was associated with a greater GRS. Because GRS evaluates the change in radial deformation, a larger GRS is an indicator of a hypercontractile left ventricle in this dimension. This finding was highly significant in the studies by Minatoguchi *et al*. [29], Celic *et al*. [27], and Cappelli *et al*. [24]. Thus a significant abnormality was present in only a small proportion of the 14 studies with GRS data.

A major finding during this review was that the reduction in GLS in hypertension was not dependent on the presence of LVH. This finding suggests that GLS is a marker for hypertensive heart disease in the absence of LVH. In contrast, GCS and GRS were not different in patients with hypertension without LVH and individuals without hypertension. The presence of LVH is indicative of hypertensive heart disease. GLS was further reduced in persons with hypertension and LVH compared to those with hypertension without LVH. This was evident in the majority (five of eight) of studies. In contrast, there were no or minimal differences in GCS and GRS for individuals with LVH compared to those without LVH. These data support the contention that GLS is an early marker of hypertensive heart disease. Furthermore it suggests that if there were only one strain marker to measure, it would be GLS.

This study suggests the concept of a continuum in the impact of hypertension on the heart. The continuum goes from a healthy person without hypertension who has a normal GLS to a person with hypertension, who because of the hypertension, has a reduction in GLS to a person with hypertension who has a further reduction in GLS to a person with hypertension who is in heart failure. Some patients travel this continuum. Clinically, a physician would not recognize the reduction in GLS so that the measurement of GLS may be a critical method to identify the beginning of the process of decline in left ventricular function.

Examining the relationship between LVEF and global strain found that there was no significant relationship between LVEF and either GLS or GCS but a significant negative correlation between LVEF and GRS. In animal studies, GLS also correlates strongly with left ventricular ${\mathrm{+dp/dt}}_{\text{max}}$ while the correlation between GRS and LV ${\mathrm{+dp/dt}}_{\text{max}}$ was weaker [50]. Which index of strain is best related to LV ejection fraction has previously been unclear but changes in GCS have been preferred. One clinical study concluded that LVEF is produced principally by circumferential shortening and is related independently to the relative wall thickness [51]. Mathematical modeling of LV contraction suggested that both longitudinal and mid-wall circumferential shortening contribute to different extents depending on the degree of abnormality of myocardial shortening [52]. Huang *et al*. [53] divided 123 patients with hypertension into 4 groups according to LVEF ranging from LVEF ≥55% to LVEF <45%. All strain measurements were reported to correlate with LVEF, with the strongest correlation with GCS and the second in GLS [53]. The most likely explanation for the difference between this meta-analysis and the study by Huang *et al*. [53] is that they examined a wide range of LVEF, so they had small subsets once the 123 patients were divided into 4 groups.

To our knowledge, this is the first meta-analysis to evaluate myocardial work indices in hypertension compared to individuals without hypertension. Each of the four indices of myocardial work showed significant differences between patients with hypertension compared to individuals without hypertension specifically GWS, GWI and GWW were increased while GWE was reduced in hypertension.

A major limitation of the use of left ventricular ejection fraction as an indicator of cardiac muscle function is the dependence of EF on both preload and afterload as well as its relative insensitivity to identify patients with early stage heart failure [54]. Assessment of LV strain minimizes the impact of preload and myocardial work adjusts for afterload.

The increase in the global work index in hypertension has been considered to be a compensatory mechanism to preserve LV contractility and function against an increase in afterload [42]. Restating this concept, an increased work demand is required to maintain adequate contractility against an increased afterload [39].

The severity of hypertension influences the magnitude of the changes in myocardial work [41, 44]. Global myocardial work index and global constructive work were higher in resistant hypertension compared to individuals with controlled-hypertension [41, 44]. Individuals with resistant hypertension have lower global work efficiency and higher global wasted work compared to individuals with controlled hypertension [40]. The concomitant presence of diabetes mellitus accentuates the effect of hypertension on myocardial work [43].

There were not a large enough number of studies that examined global work in patients with hypertension with and without LVH to perform a similar analysis that was conducted for global myocardial strain. However, it is noteworthy that Huang *et al*. [42] reported that GWI and GWW were significantly increased in hypertension with and without LVH while GWE was significantly reduced in hypertension. Comparing individuals with LVH to those without LVH, GWW was significantly increased in patients with hypertension and LVH compared with those without LVH, while GWE was significantly reduced in patients with hypertension and LVH compared with those without LVH.

### Study Limitations

The nature of meta-analysis is that it is dependent on the available published literature. The strength of the conclusions is based on the validity of each study. The available data relies on mean results from each study and does not utilize individual data from all studies. Second, while strain analysis is a relatively independent factor, it is not totally independent of other factors such as age, sex and left ventricular loading conditions [10]. Furthermore, two dimensional speckle tracking echocardiography is not without its limitations [55]. The meta-analysis examined GLS, GCS and GRS did not include assessment of strain in the various specific layers of the myocardium but rather focused on the three major global strain measurements because of their relative ease in measurement and potential extrapolation to the clinical patient care. Third, it is important to recognize that there are differences in calculated LV strain between different vendors of products to measure LV strain. This is especially an issue in the measurement of GCS and GRS which may limit the consistency in the measurement of these two factors producing greater variability so as to reduce their value. However, an analysis of the literature must be all encompassing and is unable to separate vendor specific differences from other factors such as between patient differences. The kind of LV geometry such as eccentric or concentric changes in LV mass may have a significant impact on myocardial work. However, the studies did not uniformly assess LV geometry so this factor could not be included in the assessment of LV work [56]. Lastly, in most of the analyses, in this paper, there was a considerable amount of heterogeneity between studies. A high amount of heterogeneity between studies of LV strain has been found even in controls—people without hypertension or concomitant diseases, in 24 studies considering 2597 subjects [57]. The only cause for heterogeneity in that analysis was blood pressure [57].

## 5. Conclusions

There was considerable heterogeneity between studies in some of the analyses which likely is responsible for ambiguity in the field. The strength of meta-analysis is the ability to bring the studies together and obtain an overall (group) average (‘consensuses’).

There was a significant reduction in GLS and GCS in hypertension while GRS is increased. The reduction in GLS in hypertension was not dependent on the presence of LVH. GLS, however, was further reduced in persons with hypertension and LVH compared to those with hypertension without LVH. In contrast, there were no or minimal differences in GCS and GRS for individuals with LVH compared to those without LVH. GLS is independent of LV ejection fraction. These data support the contention that GLS is an early marker of hypertensive heart disease. Global myocardial work index (GWI) and global constructive work (GCW) were significantly greater in patients with hypertension compared to controls. Global wasted work (GWW) indicated significantly less wasted work in controls compared to hypertension. In contrast, global work efficiency (GWE) was significantly lower in hypertension compared to the control.

## Supplementary Material

Supplementary material associated with this article can be found, in the online version, at https://doi.org/10.31083/j.rcm2408217.
