# Supplementary figures and images for: Assessing Myocardial Strain and Myocardial Work as a Marker for Hypertensive Heart Disease: A Meta-Analysis
Source: Rev Cardiovasc Med. 2023 Jul 31;24(8):217. doi: 10.31083/j.rcm2408217 (PMC11266759; doi:10.31083/j.rcm2408217)

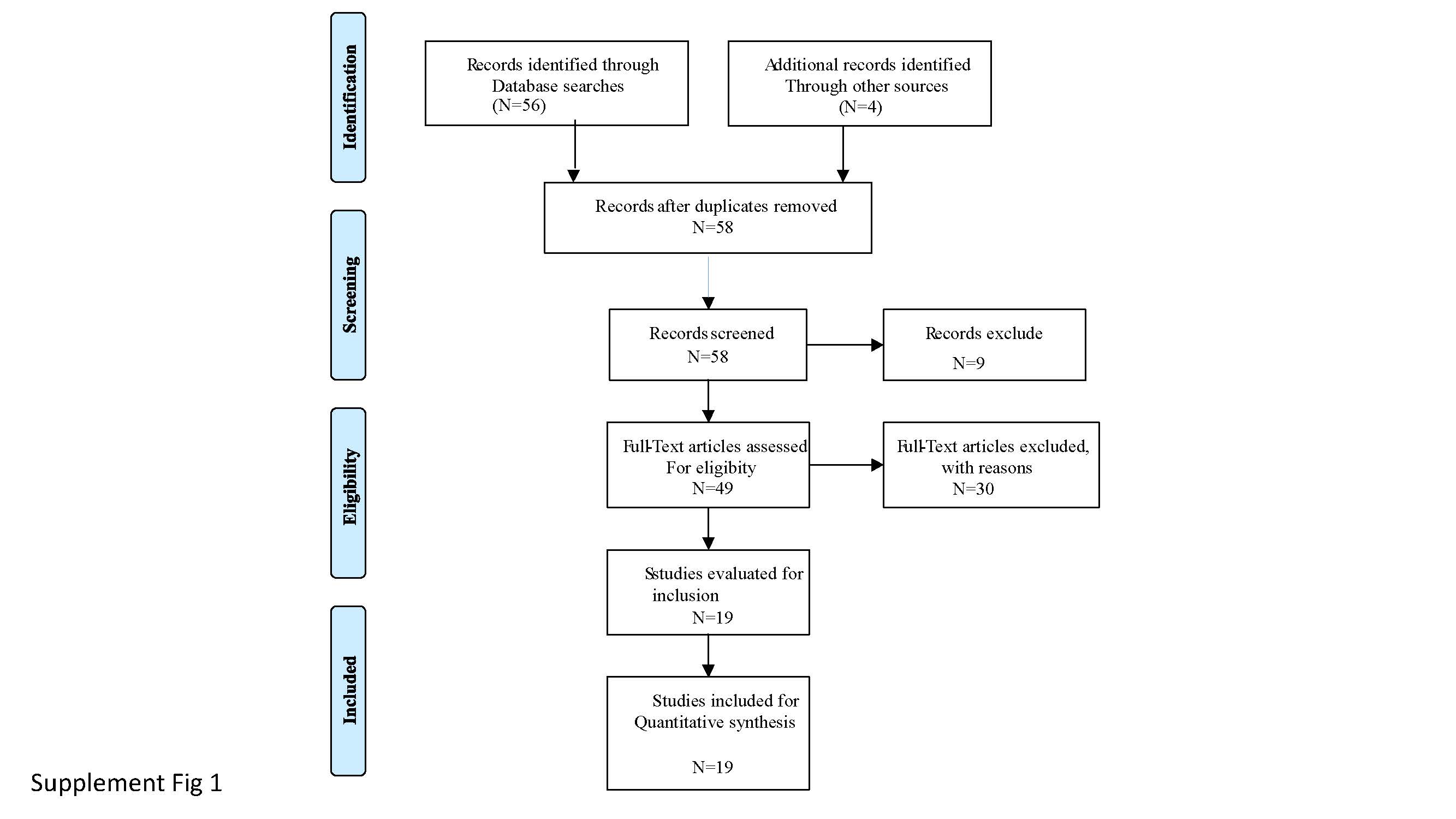

Supplement: Supplementary file 1 [file 2153-8174-24-8-217-s1.zip › 2153-8174-24-8-217-s1/supplymentary-fig1.jpg]

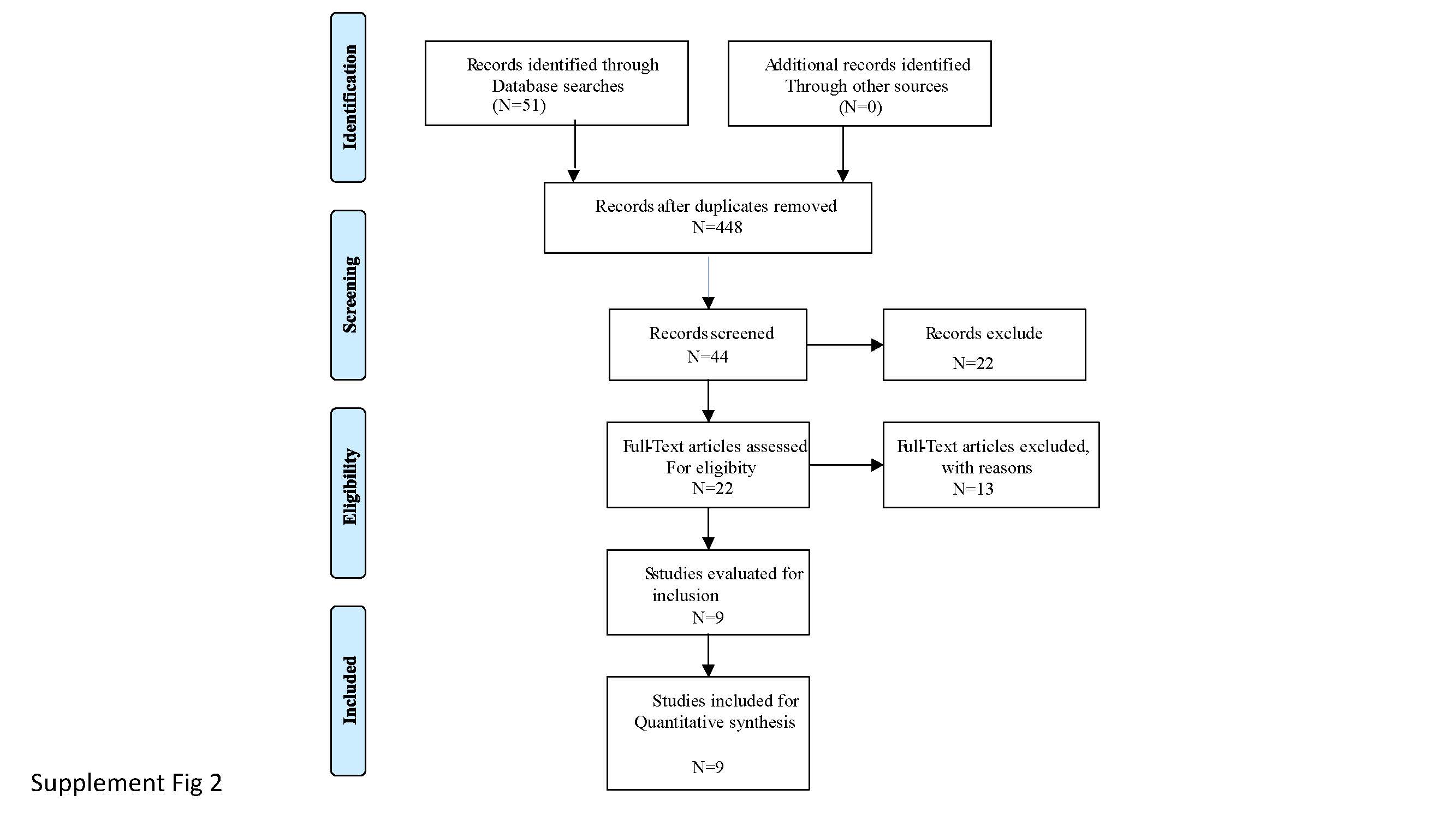

Supplement: Supplementary file 1 [file 2153-8174-24-8-217-s1.zip › 2153-8174-24-8-217-s1/supplymentary-fig2.jpg]
